# An Updated Review on SARS-CoV-2 Infection in Animals

**DOI:** 10.3390/v14071527

**Published:** 2022-07-13

**Authors:** Shujuan Cui, Yimeng Liu, Jiachen Zhao, Xiaomin Peng, Guilan Lu, Weixian Shi, Yang Pan, Daitao Zhang, Peng Yang, Quanyi Wang

**Affiliations:** Institute for Infectious Disease and Endemic Disease Control, Beijing Center for Disease Prevention and Control, Beijing 100013, China; csjbjcdc1@126.com (S.C.); lymbjcdc@126.com (Y.L.); zjcbjcdc@126.com (J.Z.); pxmbjcdc@126.com (X.P.); lglbjcdc@126.com (G.L.); swxbjcdc@126.com (W.S.); pybjcdc@126.com (Y.P.); zdtbjcdc@126.com (D.Z.); bjcdcxm1@126.com (Q.W.)

**Keywords:** SARS-CoV-2, testing, animal species, infection, strain, transmission route

## Abstract

The severe acute respiratory syndrome coronavirus 2 (SARS-CoV-2) pandemic has lasted for two years and caused millions of infections and deaths in humans. Although the origin of SARS-CoV-2 infection in humans remains unknown, infection in animals has been frequently reported in varieties of animals all over the world. Both experimental and natural infections of SARS-CoV-2 in different animal species provide useful information on viral host range and pathogenicity. As the pandemic continues to evolve, SARS-CoV-2 infection in animals will be expanding. In this review, we summarized SARS-CoV-2 testing and infection in animals as well as SARS-CoV-2 strains and transmission in animals. Current data showed that at least 18 different animal species tested positive for SARS-CoV-2. These 18 animal species belong to pet, captive, farmed, and wild animals. Fifteen of the eighteen animal species were known to be positive for the Delta variant and ten animal species were infected with two different types of variants. Human-to-animal, animal-to-animal, and animal-to-human transmission events were suggested in different outbreaks involved in animal infection with SARS-CoV-2. Continued testing, immunization, and surveillance are warranted.

## 1. Introduction

Coronavirus disease 2019 (COVID-19) was first reported in humans in Wuhan, China in December 2019, and is caused by severe acute respiratory syndrome (SARS) coronavirus 2 (SARS-CoV-2). COVID-19 causes different types of presentations ranging from asymptomatic/mild symptoms to severe illness and mortality. Common COVID-19 symptoms include fever, cough, shortness of breath, malaise, and respiratory distress. As of 5 March 2022, the SARS-CoV-2 global pandemic has caused the infection of over 443 million people and over 6 million deaths. The origin of SARS-CoV-2 remains unclear. The current evidence shows that SARS-CoV-2 could be a bat-origin coronavirus since it has the highest shared identity (96%) with a bat coronavirus RaTG13 strain [1]. Whether SARS-CoV-2 jumps directly from bats to humans or through an intermediate host remains to be determined. The animal species that are susceptible to SARS-CoV-2 also remains unclear.

*Coronaviridae* is a large family consisting of four genera, with seven human coronaviruses and varieties of animal coronavirus species. The first coronavirus discovered was the infectious bronchitis virus in chickens in the 1930s [2]. Research on coronaviruses has been increasing, with three coronavirus pandemics in humans (SARS-CoV in 2003, Middle East respiratory syndrome coronavirus (MERS-CoV) in 2012, and SARS-CoV-2 in 2020) in the past two decades. Over the past two years of the SARS-CoV-2 pandemic, several animals have tested SARS-CoV-2 positive. In this review, SARS-CoV-2, SARS-CoV-2 testing in animals, SARS-CoV-2 infection in animals, SARS-CoV-2 strains in animals, and SARS-CoV-2 transmission routes will be reviewed.

## 2. SARS-CoV-2

SARS-CoV-2 is a single-stranded, positive-sense, enveloped RNA virus. It belongs to lineage B betacoronavirus in the family of *Coronaviridae*. SARS-CoV-2 was originally identified and characterized through next-generation sequencing [3]. Different from influenza, SARS-CoV, and MERS CoV, SARS-CoV-2 has a relatively higher basic reproductive rate (R0) and transmits more efficiently in hosts [4]. Coronaviruses utilize different receptors for binding to host cells, and SARS-CoV-2 uses the same Angiotensin-converting enzyme-2 (ACE2) receptor as SARS-CoV [5]. SARS-CoV-2 is continuously mutating in hosts, and different variants such as the Alpha, Delta, and Omicron variants have emerged in the field, which raises concerns about the effectiveness of the SARS-CoV-2 vaccine [6].

## 3. SARS-CoV-2 Testing in Animals

SARS-CoV-2 testing in animals includes the detection of active infection and of previous exposure. To detect active infection, a molecular polymerase chain reaction (PCR) assay, virus isolation, and antigen tests are used [7,8]. Real-time reverse transcription PCR is commonly used in the detection of SARS-CoV-2 in animals such as cats [9,10], dogs [11], and large cats [7,8,12,13]. In addition, antigen tests and virus isolation were also used for case investigation of SARS-CoV-2 in animals [7,8]. Different from PCR with a higher sensitivity, it was shown that the antigen test has a higher specificity and produces fewer false positives [14]. Other than these routine test methods, sequencing, including next-generation sequencing, is usually utilized to characterize strains involved in the outbreaks [7,8,12].

In terms of testing previous exposure to SARS-CoV-2, the virus neutralization test (VNT), surrogate virus neutralization test (sVNT), and enzyme-linked immunosorbent assay (ELISA) have been used for the evaluation of antibody immune responses. VNT requires a biosafety level 3 (BSL3) laboratory. Different from conventional VNT, sVNT utilizes the interaction of SARS-CoV-2 receptor binding domain and ACE2, which will be blocked by specific viral antibodies in serum samples [15]. The sVNT assay skips the requirement of BSL3 and can be applied to different animal species. ELISA has been reported to determine antibody responses in animals [16,17]. A commercial double antigen multi-species ELISA was used for the detection of antibodies against the N protein for any type of susceptible animal species [16]. An in-house developed species-specific ELISA was also applied for the detection of SARS-CoV-2 antibodies [17].

## 4. SARS-CoV-2 Infection in Animals

**Figure 1:**
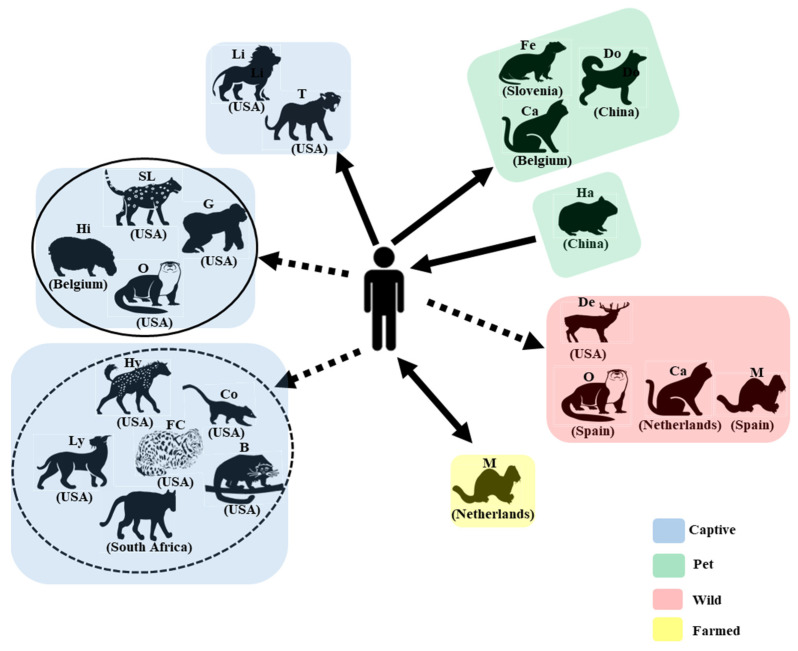
SARS-CoV-2-positive animal species are divided into four different categories: captive, pet, wild, and farmed animals. Countries in which these animals first tested SARS-CoV-2 positive are provided. Solid arrows indicate that evidence supports transmission between humans and animals, and dashed arrows suggest potential transmission. Animal species in the captive group with a dashed circle are from zoos with other animals positive for SARS-CoV-2, while those in the captive group with a solid circle are from zoos with only a single species testing positive. B: binturong; Ca: cat; Co: coatimundi; De: deer; Do: dog; Fe: ferret; FC: fishing cat; G: gorilla; Ha: hamster; Hi: Hippo; Hy: hyena; Li: lion; Ly: lynx; M: mink; O: otter, P: puma; SL: snow leopard; T: tiger.

**Figure 2:**
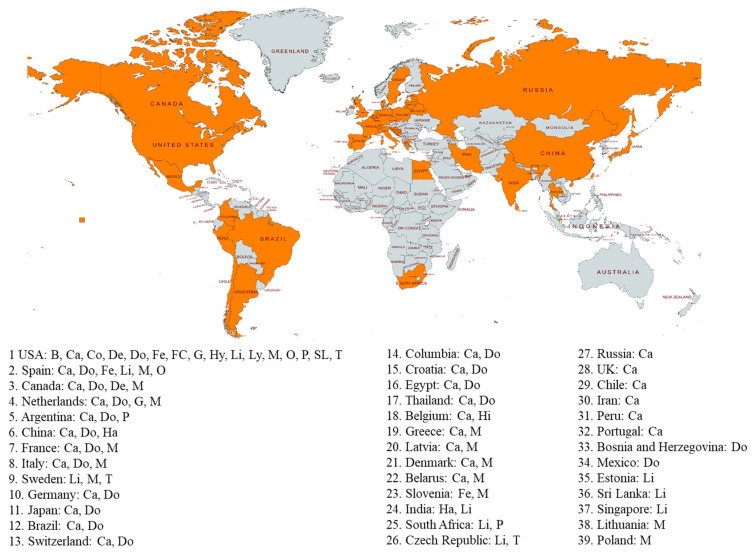
Countries with positive SARS-CoV-2 animals are marked with orange color. Animal species positive for SARS-CoV-2 in each country are provided. Until 12 May 2022, a total of 39 countries had animals that tested positive for SARS-CoV-2. B: binturong; Ca: cat; Co: coatimundi; De: deer; Do: dog; Fe: ferret; FC: fishing cat; G: gorilla; Ha: hamster; Hi: Hippo; Hy: hyena; Li: lion; Ly: lynx; M: mink; O: otter; P: puma; SL: snow leopard; T: tiger.

SARS-CoV-2 infection in animals is either asymptomatic or causes symptoms ranging from mild respiratory and gastroenteric signs to pneumonia and death. Two months after COVID-19 was reported, SARS-CoV-2 was first detected in dogs from households with positive owners in Hong Kong [11]. In March 2020, it was first reported that a domestic cat caught the SARS-CoV-2 infection, possibly through its owner who first tested SARS-CoV-2 positive in Belgium [9]. SARS-CoV-2 was detected in the nasopharyngeal swab, vomitus, and feces of the cat, and the cat developed clinical signs including vomiting, lethargy, poor appetite to anorexia, diarrhea, sneezing, coughing, and labored breathing. Following that, tigers and lions were found to be SARS-CoV-2 positive in the Bronx Zoo in New York, USA. Four tigers and three lions developed clinical signs of dry coughing and sneezing [7]. Sequence analysis suggested a potential human-to-tiger transmission. Other captive animals first found to be SARS-CoV-2 positive included a puma in South Africa in July 2020 [13]; a snow leopard (December 2020), a gorilla (January 2021), an otter (April 2021), a hyena, a fishing cat, a binturong, a coatimundi (October 2021), and a lynx (December 2021) in the USA; and hippo in Belgium in December 2021 (Figure 1). Farmed minks were first found to be SARS-CoV-2 positive in the Netherlands in April and May 2020 [18]. Since then, SARS-CoV-2 has been detected in minks in hundreds of mink farms in 14 countries (Figure 2). Other pet animals, including domestic ferrets and hamsters, also tested positive in Slovenia in November 2020 and in China in January 2022, respectively [19,20]. Other than domestic, captive, and farmed animals, SARS-CoV-2 in wild animals including deer, feral minks, feral cats, and wild otters were reported in the USA, Spain, the Netherlands, and Spain, respectively [21,22,23,24] (Figure 1). Since the first detection in these animals in different countries, SARS-CoV-2 has been detected in gorillas in the Netherlands, tigers and lions in European countries, cougars in the USA and Argentina, a domestic ferret in the USA, dogs and cats in many countries, and deer in Canada (Figure 2).

In summary, SARS-CoV-2 has been detected in 18 different animal species from ten families (Felida, Viverridae, Hyaenidae, Canidae, Mustelidae, Procyonidae, Cervidae, Hippopotamidae, Hominidae, and Cricetidae) of four animal orders (Carnivora, Artiodactyla, Primates, and Rodentia). These 18 animal species consist of pet (dog, cat, ferret, and hamster), captive (tiger, lion, snow leopard, cougar, lynx, fishing cat, binturong, hyena, otter, coatimundi, hippo, and gorilla), farmed (mink), and wild (deer, wild otter, feral mink, and cat) animals (Figure 1). The USA has 16 animal species that have tested positive for SARS-CoV-2, followed by Spain with six positive species, two countries (Canada and Netherlands) with four animal species, five countries (Argentina, China, France, Italy, and Sweden) with three positive species, 17 countries with two positive animal species (no. 10 to no. 26), and 13 countries with one positive animal species (no. 27 to no. 39) (Figure 2).

## 5. SARS-CoV-2 Strains in Animals

Similarly to other RNA viruses, SARS-CoV-2 continuously mutates in its hosts, resulting in new variants including Alpha, Beta, Gamma, Omicron, Lambda, and Mu GH. Some (Alpha, Beta, Gamma, and Omicron) are Variants of Concern (VOCs), while others are Variants of Interest (VOIs: Lambda, Mu GH) or Variants Under Monitoring (VUM: GU/490R). Based on the GISAID database [25] accessed on 12 May 2022, there are sequences available for 15 of the 18 animal species (no sequence for coatimundi, lynx, and cougar) (Table 1). Since most of animal SARS-CoV-2 cases were due to human-to-animal transmission events, strains in animals were non-variant in the earlier period, and then, variants in the latter stage of the pandemic. The Delta variant was predominantly detected in all 15 of the animal species (Table 1). Six animal species (ferret, hippo, hyena, fishing cat, and binturong) were only infected by the Delta variant, whereas ten animal species (cat, dog, mink, deer, tiger, lion, snow leopard, gorilla, hamster, and Otter) were infected by more than one type of variant, and eight animal species (dog, cat, mink, deer, tiger, lion, snow leopard, and gorilla) were also infected by non-variant strains. Cats were infected by five variants: Alpha, Delta, Gamma, Lambda, and Omicron; deer were infected by four variants: Alpha, Delta, Gamma, and Omicron; dogs and minks were infected by Alpha, Delta, and Omicron; tigers, lions, gorillas, and otters were infected by the Alpha and Delta variants; hamsters were infected by the Delta and Omicron variants; and snow leopards were infected by the Delta and Mu GH variants (Table 1).

## 6. SARS-CoV-2 Transmission Routes

Since the first case of animal infection with SARS-CoV-2 in 2020, much effort has been put into tracking the infection sources in animals, which is the key to controlling infection in them. Several lines of evidence suggest that infection in dogs [11,16,26,27], cats [9], large cats [7,8,12,13], and domestic ferrets [19] were due to human-to-animal transmissions (Figure 1). In the case of the farmed minks, it was reported that minks were infected through a human-to-mink transmission route [18]; then, infected minks transmitted the virus back to humans [28] (Figure 1). Infected hamster-to-human transmission has also been suggested in Hong Kong [29] (Figure 1). Although the exact mechanism by which deer caught the SARS-CoV-2 remains unclear, the findings from one previous study indicated that human-to-deer and deer-to-deer transmission events could occur [21]. Animal-to-animal transmission events in other animal species are highly possible as long as two or more animals are involved in the outbreaks. Feral cats testing positive for SARS-CoV-2 could contract it through mink-to-cat or cat-to-cat transmission events [23]. Unlike infections in pet, captive, and farmed animals, it is more difficult to trace and control infections in wild animals, and how these wild animals caught the virus remains unclear. These wild animals (deer, mink, and otter) could catch the virus through a contaminated environment (such as water) and close contact with domestic animals and humans.

## 7. Conclusions

SARS-CoV-2 infection in animals has become complicated since different types of animals have tested positive for SARS-CoV-2. There are different strategies used to control infections in animals and humans, including culling farmed animals, the isolation of infected pet and captive animals, and the vaccination of captive animals. In 2021, lots of zoo animals were immunized with a recombinant vaccine donated by the Zoetis company. There is also a commercial SARS-CoV-2 animal vaccine, Carnivac-Cov, available in Russia. This vaccine is designed for carnivores [30]. Surveillance of SARS-CoV-2 in wild animals has been conducted in Europe and America [21,22,23,24,31], and the control of SARS-CoV-2 infection in wild animals is quite challenging. A possible approach for controlling SARS-CoV-2 in wildlife is the application of an oral vaccine to them, which is similar to the strategy used to control rabies in wildlife.

## Figures and Tables

**Table 1:** Number of sequences of variant and nonvariant strains of SARS-CoV-2 in 18 animal species. GISAID SARS-CoV-2 sequence database was accessed on 12 May 2022. NA: not available; Non-Var: non-variant.

Species	Total	Alpha	Delta	Gamma	Omicron	Lambda	MU GH	Non-Var
Cat	140	11	27	1	5	3		93
Deer	159	3	38	1	4			113
Mink	1366	6	60		2			1298
Dog	84	5	24		7			48
Lion	74	3	42					29
Tiger	43	3	27					13
Gorilla	15	2	12					1
Otter	8	5	3					
Hamster	24		12		12			
Snow Leopard	9		4				4	1
Binturong	1		1					
Hyena	1		1					
Ferret	1		1					
Hippo	1		1					
Fishing Cat	1		1					
Lynx	NA							
Cougar	NA							
Coatimundi	NA							

## Data Availability

The data presented in this manuscript are available in Figure 2 and Table 1.
